# Ototoxicity management: An investigation into doctors’ knowledge and practices, and the roles of audiologists in a tertiary hospital

**DOI:** 10.4102/sajcd.v63i1.174

**Published:** 2016-12-01

**Authors:** Anna Wium, Berna Gerber

**Affiliations:** 1 Centre for Medical Ethics and Law, University of Stellenbosch, South Africa; 2 Devision of Speech-, Language- and Hearing Therapy, University of Stellenbosch, South Africa

## Abstract

**Background:**

A significant number of medications that are prescribed by doctors to treat cancers, tuberculosis and infections are ototoxic. Disclosure of ototoxic risks is ethical practice as patients have the right to be properly informed about and involved in decisions about their health care. Often, doctors fail to disclose such information.

**Aim:**

This research investigated whether a group of doctors working in a South African academic hospital inform their patients about the ototoxic risks associated with specific medications, and if not, explore the reasons for it. It was determined what the participants’ knowledge levels of ototoxicity were as knowledge is seen as a precursor to disclosing information to their patients. A further aim of the research was to determine whether audiologists should expand their role by sharing information with patients and other professionals in the management of ototoxicity and in the hospital.

**Method:**

There were 90 participants included in the study through convenience sampling, which represented interns, medical officers, registrars and consultants in the neonatal intensive care unit, intensive care unit, ear–nose–throat, and internal and family medicine departments. The research made use of a descriptive survey design that collected mainly quantitative data and a limited amount of qualitative data through questionnaires. The data were descriptively analysed, and the qualitative data were listed and quantified.

**Results:**

The research firstly determined the participants’ knowledge and understanding of ototoxicity, and it was found that there was room for improvement. With reference to the current practices of doctors in the prescription of ototoxic medicines, it was found that disclosure of ototoxic risks was limited, mostly because of a lack of time and insufficient knowledge. In comparing knowledge and practices between levels of employment, it was found that particular post levels performed better than others. The participants regarded the role of the audiologist as team member important, although very few referred their patients for audiological monitoring when they prescribe ototoxic medication.

**Conclusion:**

A need for additional support to doctors was identified, which indicates that audiologists should expand their role to include the provision of continued professional development activities and to renew their efforts to advocate their role in the hospital so that doctors are made aware of the importance to refer their patients for ototoxic screening and monitoring.

## Introduction

Much progress has been made in terms of clients’ rights within the health care environment. Such changes place the actions of health care professionals under an ethical magnifying glass and require revisiting the concept of professionalism. Medicine is regarded as a profession, and it implies ‘… a commitment to serving the public so that patients^1^ receive the benefit of health’ (Moodley, 2013b, p. 4). When considering the fiduciary relationship that exists between health care professionals and patients, professionalism can be seen as ‘… acting out the values and beliefs in individuals who serve those whose well-being is entrusted to them by putting the client’s interests first’ (Kirk, 2007, p. 13). It is important that each individual within a health care team acts responsibly and in the patient’s best interest, and see to it that their colleagues also do so (Moodley, 2013a). Health care professionals’ duties towards their patients include providing patients with the necessary information, in a manner that the patients can understand, so that patients can exercise their right to autonomous decision-making about the health care they receive (Beauchamp & Childress, 2013).

This research investigates informed consent and disclosure of important information in the management of ototoxicity. It aims to determine what doctors’ knowledge of ototoxic medication is, and whether doctors as a rule disclose ototoxic risks to their patients and, if not, to find the reasons for their not doing so. Knowledge is seen as a precursor to disclosing such information to their patients. Should it be determined that the participants had insufficient knowledge of ototoxicity to adequately inform their patients about the risks, the aim of the research was to determine whether audiologists, as part of the health care team and in a supportive role, should expand their role in the management of ototoxicity to do so. The research, therefore, falls within the field of descriptive ethics as it investigates the moral behaviour of a group of doctors working in a tertiary hospital.

A significant number of medications that are prescribed by doctors to treat cancers, tuberculosis (TB) and infections cause damage to the inner ear, and therefore are ototoxic (Schellack & Naude, 2013). Population groups at risk for ototoxic treatments include patients suffering from infections, those in oncology units, patients with renal failure and newborn babies in neonatal intensive care units (NICU) (Engler, Schellack & Naude, 2013). In most cases, patients who are admitted to hospital wards are extremely sick and are desperate to have their conditions treated. This desperation for better health places them in a vulnerable position where they often unconditionally accept the doctor’s advice regarding how their condition should be treated. They trust that their doctors know and will do what is best for them (Moodley, 2013b) and do not necessarily question the risks attached to their treatment. As a rule, doctors should select the most cost-effective and least toxic medication necessary to effectively treat their patients. Doctors have an ethical and professional duty to disclose the potential harm of medication (including ototoxic medication) that they prescribe and to obtain informed consent from patients prior to treatment. Should doctors not disclose information (such as adverse effects of medicines), it can be considered as unethical practice. The problem of insufficient patient involvement in decision-making can be exacerbated in contexts such as South Africa, where linguistic and cultural divides between health care professionals and patients are common. Furthermore, patients with low levels of education and literacy are vulnerable to have their right to autonomous decision-making compromised. Sadly, there are many such patients who make use of the public health care service in South Africa.

## Background

With the focus of this study on ototoxicity and informed consent, it is necessary to address some key concepts, namely the nature of ototoxicity and ototoxic medication as well as the management of ototoxicity from an ethical perspective. This includes a discussion on the role of the audiologist in the management of ototoxicity.

### Ototoxicity

Patients at risk of the effects of ototoxicity (Dobie, Black, Pezsnecker & Stallings, 2006) typically include those receiving treatment for TB, those in oncology units or those who are suffering from renal failure. Newborn and/or premature babies who are put on such medication in NICU may also be at risk. Patients most likely to develop ototoxicity-related adverse effects appear to have a genetic tendency because of a mutation in the mitochondrial RNA in cells of the organ of Corti (Banotai, 2004).

Roland and Rutka (2004) described ototoxicity as the tendency of certain substances, which are administered either in a systemic or topical manner, to cause functional impairment and cellular damage to the tissue of the inner ear, especially to the end organs of the cochlear and vestibular divisions of the eighth cranial nerve. Ototoxic drugs (aminoglycosides and antineoplastic drugs) can create damage to the basal region of the cochlea, which will affect the perception of high-frequency sounds and later can also affect the apex of the cochlea where the perception of low-frequency sounds originate from (Schellack & Naude, 2013). Such medication could cause permanent sensorineural hearing loss, tinnitus and/or disequilibrium, as well as difficulty to understand speech in background noise.

The damage can present as either cochlear toxicity (damage to the cochlea) or vestibular toxicity (damage to the vestibular system) or both (Selimoglu, 2013). Cochlear toxicity can result in sensory neural hearing loss with permanent tinnitus and hyperacusis. Patients may find it difficult to discriminate voice from background noise. When the vestibular system is damaged, patients present with disequilibrium, ataxia, nystagmus, oscillopsia and vertigo (Selimoglu, 2013). Although sources differ, it is estimated that aminoglycosides affect approximately 33% of patients (Cheng, Huth & Ricci, 2011), and chemotherapy drugs (e.g. cisplatin or carboplatin) affect 22%–70% of patients (Banotai, 2004; Yansey *et al*., 2012).

Ototoxic drugs include antibiotics (e.g. gentamicin, tobramycin and amikacin), loop diuretics (e.g. furosemide) and platinum-based chemotherapy agents, such as cisplatin (Hellberg *et al*., 2009). Other ototoxic medication includes macrolide antibiotics, quinine derivatives and also nonsteroidal anti-inflammatory drugs, which could cause temporary hearing loss that is reversible (Professional Board for Speech Language and Hearing Professions, 2015).

The probability of ototoxicity can be decreased if the administration of aminoglycosides is monitored using pharmacokinetic principles (Banotai, 2004). Aminoglycoside-related ototoxicity, as well as that caused by platinum-based chemotherapy agents, is irreversible (Selimoglu, 2013). The ototoxic effect of loop diuretics (e.g. furosemide and ethacrynic acid), macrolide antibiotics (e.g. clarithromycin and erythromycin) and antimalarial drugs (e.g. chloroquine and quinine) is temporary and/or reversible. It is, however, possible that a pre-existing hearing condition was present before taking the ototoxic drugs, which may then cause an additional high-frequency hearing loss or exacerbate an existing high-frequency hearing loss. Adequate hearing from birth onwards is essential for language development and later literacy development (Paul & Norbury, 2012). The impact of hearing loss on the quality of life is detrimental (Konrad-Martin *et al*., 2005) and, therefore, should be prevented as far as possible through careful monitoring and management of ototoxic medication.

### Management of ototoxicity from an ethical perspective

Ethical behaviour is expected from health care professionals, at least since the time of ancient Greek physician Hippocrates, who advocated the principle of *primum non nocere* (Moodley, 2013a, p. 5). Finally, the emphasis in healthcare ethics is on patients’ rights, as well as teamwork (Naude & Bornman, 2014). Patients have the right to be informed about their health care options, particularly if there is a risk of serious side effects such as hearing impairment or potential deafness.

A decision in favour of ototoxic treatment based on the net benefit of treatment fits in well with the principlism approach to bioethics, which emphasises the four ethical principles of respect for autonomy, beneficence, non-maleficence and justice. Within the principlism approach (Van Niekerk, 2013), these principles are not viewed as absolute but rather as *prima facie*, meaning that they need not and cannot be followed without exception. In deciding which course of action to follow in a specific situation, the relevant principles and the rules stemming from them should be weighed and balanced, and the principle(s) that carry the most weight (from a moral standpoint) in the particular circumstances should be adhered to. When discussing ototoxicity from an ethical perspective, the focus is particularly on the principles of respect for autonomy, beneficence and non-maleficence.

#### Respect for patient autonomy

The bioethical principle of respect for patient autonomy creates four obligations for health care professionals, namely informed consent, confidentiality, truth telling and communication (Moodley, 2013c). Informed consent includes the process of providing the necessary information to patients about their health care options, particularly when there are risks involved (such as hearing impairment or potential deafness), so that they can make informed choices about the course of action to follow. It is the right of the patient to receive a sufficient explanation of his or her condition, the possible treatment options, the risks and benefits associated with each of these options, and the prognosis without treatment. The process of informed consent also requires engagement with the patient before a treatment decision is reached. Informed choices are dependent on the nature and quality of the information provided. According to South African law, such choices should be exercised by adults (> 18 years), and whenever children or adolescents (< 18 years) are involved the parent or guardian must be informed about the risks of treatment (South African Government Gazette, 2005). Children >12 years can provide consent to their own medical care, provided they have the mental capacity to understand the risks, benefits and other implications of the treatment (South African Government Gazette, 2005).

Beauchamp and Childress (2013) described the elements of informed consent: informed consent to an intervention can be provided only when one is competent to act and has received a thorough disclosure of core information and comprehend such a disclosure. Once the information has been provided, it is necessary to assess the patient’s understanding thereof. Accompanying the disclosure should be a recommendation of a plan of action (including alternatives), which can then be accepted or rejected by the patient. Patient’s consent to the intervention should be voluntarily.

#### Beneficence and non-maleficence

Beneficence refers to actively promoting and seeking what is in the patient’s best interest and considers the patient’s pain, and the risk of disability and death (Emanuel *et al*., 2008). Closely related to the principle of beneficence, non-maleficence refers to the obligation to ‘do no harm’. In the case of ototoxicity, the risk of hearing impairment can be seen as a harm that is outweighed by the greater harm of the risk of death or serious disease. The benefits (avoidance of death and possible recovery) outweigh the potential harm of treatment (e.g. hearing and/or vestibular impairment).

Doctors’ ethical reasoning is often heavily influenced by deontology and utilitarianism, which both consider the question: ‘What is the right thing to do?’. When doctors prescribe ototoxic medication, their decisions mostly are based on a consequentialist view where the morality of the decision stems from the expected outcome or result (healing and avoidance of death) (Van Niekerk, 2013). In the case of ototoxicity, the one extreme can be seen as a matter of ‘death versus deaf’. Such an approach aims to be impartial and does not consider aspects such as attachment in special relationships or concern for those towards whom one has a specific social role as important in ethical reasoning and decision-making. There is no room for special circumstances and relationships in such rule-based decision-making processes. A theory which values the role of relationships, emotions and special circumstances in making decisions is the ethics of care (EoC), which will be discussed in the following section.

### The ethics of care

Gilligan (1982) was of the opinion that the emotions and certain character traits, including the capacity for sympathy, form an important part of ethical decision-making, a perspective that is referred to as the EoC. The EoC is suitable for the clinical health care context because the health care professional (e.g. the doctor and the audiologist) should be responsive to the patients’ and their caregivers’ needs, and show concern for them. It is well suited to deal with decision-making and difficult discussions within health care, such as disclosures, which typically involves participation of the patient’s family with the health care team in a supportive role (Gerber, 2013). The roles of the audiologist (Health Professions Council of South Africa, 2005) resonate well with such a perspective.

The EoC perspective developed from virtue ethics and is the moral perspective most closely identified with modern feminist philosophy (Van Niekerk, 2013). The contention is not that the rule-based traditional moral theories are erroneous, but rather that they account for *only a part* of the whole moral world. Prominence is given to care within personal relationships and specific circumstances. Patients who are very sick, are prescribed ototoxic medication and are at risk for hearing loss find themselves in a vulnerable position and are in need of emotional and informational support. From an EoC perspective, the management of such a patient turns the focus from *what* one should do (the right action) to *how* the action should be performed (emphasis on the relationships and the role of the emotions in morality). There is, thus, a difference in emphasis rather than a difference in primary values. The EoC perspective thus supplements rather than replace the traditional theories.

The significance of care within the audiologist–patient or audiologist–client relationship that stems from their professional roles empowers audiologists to not only treat the disorder (scientifically) but also attend to the psychosocial aspects of the patient’s situation (Naude & Bornman, 2014). Such an approach favours both the audiologist and the patient.

### Audiologists and ototoxicity

Professional ethics call for a commitment to patients and to other health care professionals. Audiologists are bound by their professional code of ethics, which relies on the four principles of common morality (Van Niekerk, 2013): respect for autonomy, beneficence, non-maleficence and justice. The roles of audiologists (Stach, 2010) include the prevention, identification, assessment, diagnosis, rehabilitation, counselling, advocacy or consultation, as well as education, research and administration related to hearing and balance disorders. Such roles are nurtured throughout their professional training with the main focus on the development of professionalism.

In terms of ototoxicity management, a much narrower role has been described for the audiologist by the Professional Board for Speech Language and Hearing Professions (2015), which specify assessment of baseline hearing sensitivity prior to treatment, the monitoring of hearing at regular intervals, management of hearing impairment, as well as vestibular assessment and management (Professional Board for Speech Language and Hearing Professions, 2015). Although these roles are essential in the management of ototoxicity, they may need to be revisited to accommodate more of a supportive role in addition to their existing roles.

Although audiologists are bound by the four principles of bioethics (Van Niekerk, 2013), the nature of their work enables them to develop partnerships with their clients. An EoC view on morality is, therefore, also suitable when managing ototoxicity and hearing loss.

Ototoxicity can impact negatively on communication, coping skills and quality of life. It is, therefore, of crucial importance that the ototoxic effect of medication be determined as soon as possible (Konrad-Martin *et al*., 2005). Audiologists have an important role in monitoring ototoxicity that affects cochlear and vestibular function (e.g. aminoglycoside or cisplatin ototoxicity) (Professional Board for Speech Language and Hearing Professions, 2015).

Monitoring of ototoxicity is performed through a series of hearing tests, which include basic audiometry, high-frequency audiometry, oto-acoustic emissions, automated auditory brainstem responses, vestibulotoxicity monitoring and the Dizziness Handicap Inventory (Hall, 2000). Such monitoring should continue even after patients have stopped taking the ototoxic medication. From bedside tests, they can determine adverse effects that affect the hearing and balance and notify the doctor immediately. Any changes in the cochlear function of the patient are likely to be first detected by the audiologist and should be reported to the patient, his or her family and the other members of the health care team (Katz, Medwetsky, Burkard & Hood, 2009).

Early identification of hearing loss allows for timely counselling of the patient and his or her significant others (Professional Board for Speech Language and Hearing Professions, 2015). Counselling of patients with hearing loss as a result of ototoxic medication implies that audiologists provide patients and their families with information on the symptoms of ototoxicity, side effects of certain medicines, otoprotective strategies and management of hearing loss (which may include fitting the client with a hearing aid) in order to help the patient make informed decisions about the treatment options should he or she present with hearing loss. Patients have a right to be informed of the possible side effects of their prescribed medicine and the management of ototoxicity. Audiologists also have an educational role to play towards other members of the health care team in terms of providing information and training on relevant topics (e.g. hearing loss and ototoxicity).

### Ototoxicity and informed consent

Patients have a right to make informed decisions regarding their health and treatment. Some prescribed medications present an ototoxic risk to patients. Although it is expected that professionals will act responsibly and to the benefit of their patients, doctors may not necessarily inform their patients about the potential risks of certain medicines.

In the case of ototoxicity, ethical practice requires that patients be made aware of the name of the drug, the dosage and administration and how it is absorbed and excreted, the kidney and liver function and the potential risks for ototoxicity. Not all hospitals have such protocols and services in place. In addition, doctors may tend to focus more on treating the primary pathology than the patient holistically (De Andrade, Hajat & Khoza-Shangase, 2009).

This then brings one to the question as to what the role of the audiologist should be in this instance. Considering the roles of audiologists (Health Professions Council of South Africa, 2005), the management of ototoxicity falls within their scope of practice (Stach, 2010). Should audiologists not take it upon themselves to provide more patient-centred care to patients and disclose information on ototoxicity? During their training, audiologists are encouraged to provide patient-centred services which entail aspects of respect, provision of emotional support and physical comfort, disseminating information through effective communication (that also includes the involvement of family and carers), care coordination and access to care (Gerteis, Edgman, Levitan, Daley & Delbanco, 1993).

## Methodology

### Aim and objectives of the research

The aim of this research was to determine what doctors’ practices were with regard to the prescription of ototoxic medications and what their perceptions were regarding the role (and potential role) of audiologists in the management of ototoxicity at a tertiary institution in a semi-rural context. The objectives of the research were as follows:

- To determine participants’ knowledge and understanding of ototoxicity (including knowledge of side effects and ototoxic compensatory strategies).
- To explore the current practices of doctors when prescribing ototoxic medication, which addresses participants (non-participants) or disclosure of side effects of ototoxic medicines and the reasons for not disclosing, referral of patients to audiologists for ototoxicity monitoring, and the use of resources on ototoxic medicines.
- To determine how the participants regard the role of the audiologist in terms of ototoxicity management in the hospital.

The context of the research was a tertiary hospital in a township context, which is in a previously disadvantaged context in South Africa where unemployment is high, and poverty with associated medical conditions (e.g. AIDS and TB) are endemic. Patients often have low education and literacy levels. In this hospital, doctors from all levels of employment have large workloads and have to deal with challenges inherent to the public health system.

A purposive and convenience sampling method was used as all doctors who were present at the academic or departmental meeting of specific departments on a given day were included. The research sample (Table 1) included 90 participants consisting of interns (*n* = 14); medical officers (MOs) (*n* = 14); registrars (Reg) (*n* = 38) and consultants (*n* = 24) within the departments of internal medicine (Internal Meds), intensive care unit (ICU), ear–nose–throat (ENT) surgery, NICU, paediatrics (Paeds) and family medicine (Fam Med).

**TABLE 1:** Description of the research sample.

Department or ward	Intern		MO		Reg		Consult		Number
	%	*n*	%	*n*	%	*n*	%	*n*	
Internal Med	19	5	15	4	27	7	38	10	26
ICU	0	0	14	1	29	2	57	4	7
ENT	11	1	11	1	67	6	11	1	9
NICU	0	0	50	1	50	1	0	0	2
Paeds	13	2	25	4	50	8	13	2	16
Fam Med	20	6	10	3	47	14	23	7	30
Total	-	14	-	14	-	38	-	24	90

ENT, ear–nose–throat; Fam Med, family medicine; ICU, intensive care unit; Internal Med, internal medicine; MO, medical officers; NICU, neonatal intensive care unit; Paeds, paediatrics, Reg, registrars.

The study made use of a survey design, which collected mainly quantitative data. Questionnaires (Appendix A) consisting of both closed-ended and open-ended questions were used to collect the data from doctors in these specific units or departments. The self-constructed questionnaire consisted mainly of closed-ended questions, checklists and questions with Likert-type scale answers. Only a limited number of open-ended questions were included. The questionnaires were designed to be user-friendly and to allow for quick and easy completion. The questions were reviewed by two experts in questionnaire design (of which one was also an expert in pharmacology). The questionnaires were approved for use by a research and ethics committee (S14/04/082).

Permission to conduct the research and ethical clearance was obtained from a research and ethics committee, as well as the management of the specific hospital. A pilot study was conducted where three doctors, who were not participants, acted as critical readers and completed the questionnaires to report on the clarity of the questions, the length of the questionnaire and the time required for completion prior to use. Based on the results of the pilot study, changes were made to the layout of the questionnaire and numbering, as well as the question format of one question.

The various department heads were informed of the study, who in turn arranged for the researcher to address the staff during an academic or routine morning meeting in their respective departments. The participants were verbally informed about the nature of the study, and informed consent was obtained prior to distributing the questionnaires (Appendix A). Questionnaires were completed anonymously and afterwards the participants were offered muffins as a gesture of goodwill to thank them for their time. The participants placed the completed questionnaires in a box at the door of the venue when they left, and these were collected by hand afterwards.

Completed questionnaires were scored according to model answers that were obtained from the literature. Qualitative data for each open-ended question were listed in excel format and coded. All codes were listed and grouped into categories. The demographic information was correlated with the results obtained from the other sections (e.g. relationships were obtained between the participants’ performance on knowledge-related questions and the resources they accessed). Qualitative data were also quantified by being scored on a binary scale (Creswell, 2008). All the data were analysed descriptively by calculating averages and percentages in excel. Data obtained from both quantitative and qualitative data were triangulated through corroboration to deduct conclusions. Results were presented as graphs and tables.

An effort was made to adhere to all ethical principles when conducting the research. By obtaining informed consent and emphasising that participation was voluntarily, the researchers based the research on the ethical principle of autonomy. The data were collected anonymously and participants’ information was treated with confidentiality adhering to the principle of respect.

## Results and discussion

The results are presented and discussed in accordance with the research objectives. The research firstly addressed the participants’ knowledge of ototoxicity, particularly on the various side effects, and otoprotective strategies. Secondly, the research determined their current practices in ototoxicity management, for example, disclosing side effects, otoprotective strategies, as well as resources consulted in this regard. Lastly, the role of the audiologists with regard to ototoxicity management in the hospital as regarded by the participants is also explored (e.g. whether they consider them as members of the ototoxicity management team, ototoxicity monitoring, disclosing of ototoxicity risks and support to patients who develop hearing loss as a result of ototoxicity).

### Objective 1: Participants knowledge of ototoxicity

In determining the participants’ knowledge of ototoxicity, the research focussed on whether the medicines were ototoxic or vestibulotoxic, the risk factors as well as the most suitable otoprotective strategies to be used in the management of ototoxicity.

### Participants’ knowledge of side effects

The participants were required to indicate from a list of medicines which medicines were either cochleotoxic or vestibulotoxic, and to indicate the risk factors for ototoxicity. The results obtained from questions on all these aspects were analysed and are presented in Table 2.

**TABLE 2:** Participants’ knowledge of ototoxic medicines and their side effects.

Number of participants	Scores (%)	% of participants
1	30	15.5
6	40	
3	45	
4	50	
3	55	31
12	60	
5	65	
3	70	
5	75	
22	80	53
9	85	
6	90	
5	95	
6	100	

Table 2 shows that the majority of the participants (53%, *n* = 48) achieved a score of ≥80%, which implies them to have adequate knowledge of ototoxic medicines and that 47% will benefit from additional information and increased knowledge to various degrees. When the participants’ knowledge of ototoxicity was further explored and was compared across various departments (Table 3), it can be seen that there is a difference. It is acknowledged that such a comparison should be viewed with caution as the sample size does not allow for conclusions based on statistical significance. However, such results suggest that knowledge of ototoxicity could be context specific, as some departments scored higher than others. The variance in these results indicates that additional support (e.g. continued professional development (CPD) activities) with regard to ototoxicity should be provided to specific departments.

**TABLE 3:** Knowledge of side effects of ototoxic medicines compared across various departments.

Department	Average scores (%)
Internal medicine	78
ICU	78
ENT	58
NICU	80
Paeds	67
Family medicine	63
Total	73

ENT, ear–nose–throat; ICU, intensive care unit; NICU, neonatal intensive care unit; Paeds, paediatrics.

The participants had varying opinions with regard to otoprotective strategies^2^ (Table 4), from which deductions can be made with regard to their knowledge in this matter.

**TABLE 4:** Participants’ opinions of otoprotective strategies (quantitative data).

Variable	No answer (%)	Disagree (%)	Agree (%)	Not sure (%)
No time	12	50	14	23
Time limits	17	46	17	21
Waste of time	13	72	2	12
Effective	10	4	62	23
Support use	8	3	86	3
Need more info	6	3	89	2

The results presented in Table 4 show that there was 89% of the participants who agreed that they needed more information on otoprotective strategies, which indicates that these participants did not feel confident in terms of their knowledge on this matter. From Table 4, it is also evident that 86% of the participants agreed that they support the use of otoprotective strategies. It thus seems that they knew what the right thing was to do but were unable to do so in practice. It can be assumed that a person will be hesitant to disclose information on otoprotective strategies if they do not feel confident about his or her knowledge in this regard.

**FIGURE 1:**
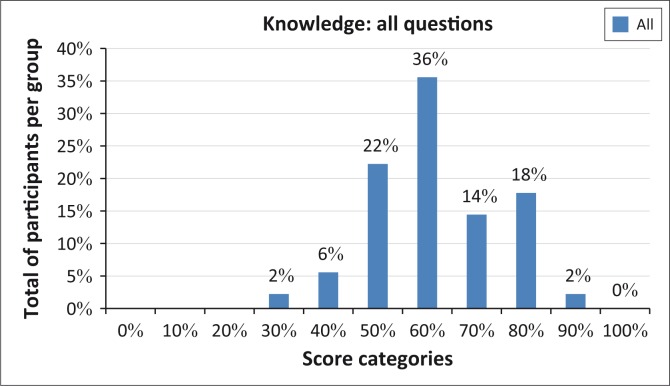
Comparison of participants’ knowledge of ototoxicity as measured by all questions (for the entire group of participants).

In summary, when the results of all questions which determined knowledge of ototoxicity were analysed for the group, it showed a peak at 60%–70% (Figure 1).

**FIGURE 2:**
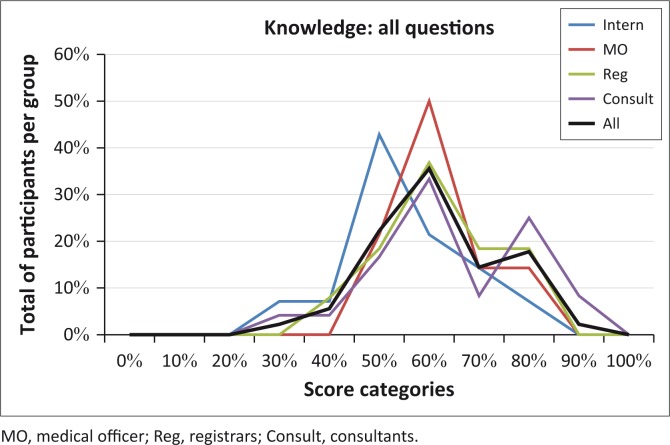
Comparison of participants’ knowledge of ototoxicity as depicted from all questions across positions.

A similar analysis was performed across the various levels of appointment on responses to all questions that explored knowledge of ototoxicity and compared performance across levels of appointment (Figure 2).

The results (Figure 2) show that 50% of all MOs obtained scores of 60%, and 35% of the entire group scored around 60% for the knowledge section in the questionnaire. The consultants show a dual peak, whereas the others typically have lower scores than consultants. See also the ‘bottle graph’ phenomenon, which shows a slightly higher score for consultants than others, and some growth opportunities for the interns, which can probably be ascribed to more experience and higher levels of education within the rest of the participant group. Educational background and/or years of experience probably have an influence on their performance. Consultants with more experience and higher levels of educational credentials are instrumental in the training of junior doctors and can, therefore, be expected to be more knowledgeable. They also play an important quality control role in the hospital.

**FIGURE 3:**
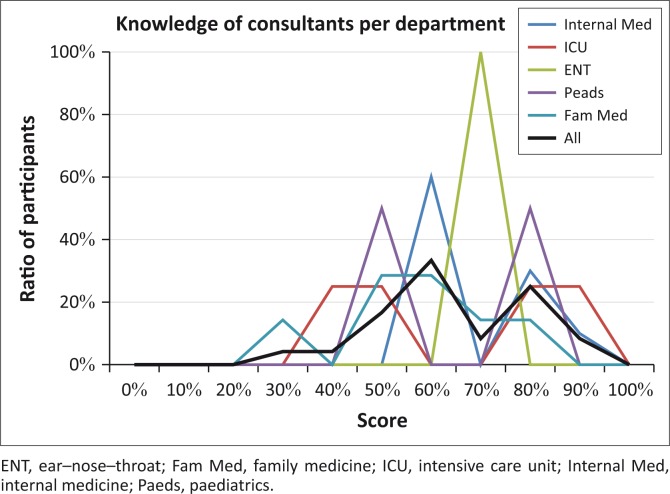
Comparison of consultants’ overall knowledge of ototoxicity across departments.

When the knowledge of ototoxicity of the consultants was investigated across departments (Figure 3), it shows that the paediatrics department presents with dual peaks. Insufficient data are available to explain this fact, but it is postulated that consultants in some departments were more knowledgeable with regard to ototoxicity than those in others. It seems as if knowledge of this specific topic is context (department) specific. The findings obtained in terms of knowledge revealed that doctors do not necessarily have adequate knowledge of ototoxicity symptoms or treatment, which is consistent with results reported by Khoza-Shangase (2013) and De Andrade et al. (2009). In essence, those participants with lesser knowledge could benefit from CPD activities that focus on the management of ototoxicity. CPD activities can, thus, be tailor-made to meet the needs of for specific departments.

**FIGURE 4:**
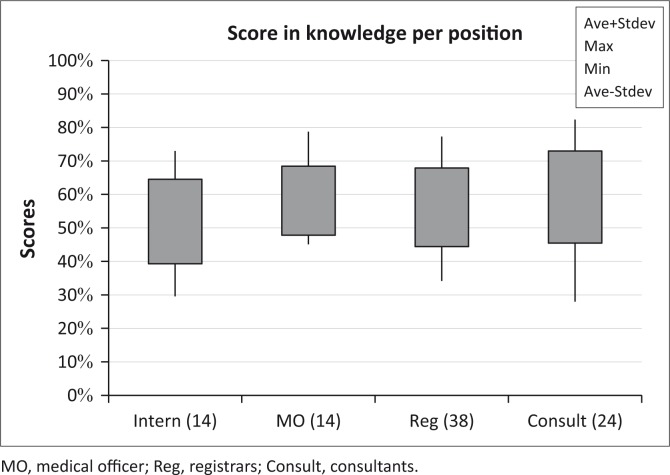
Knowledge of ototoxicity per position.

Figure 4 shows the knowledge of all the participants across the various positions.

With reference to Figure 4, there is not much difference in knowledge of the participants overall, although consultants score highest and interns lowest. This probably relates to years of experience and knowledge accrued over time, as well as their credentials. It is clear that there is room for improvement of knowledge on ototoxicity across all levels of appointment, and additional information should be provided to them.

By corroborating the quantitative and qualitative results on the participants’ knowledge of ototoxicity, it can be concluded that the participants need additional training in ototoxicity (e.g. the types of medicines that are ototoxic, what the side effects are, as well as otoprotective strategies).

### Objective 2: Current practices of doctors when prescribing ototoxic medicines

The participants’ current practices when prescribing ototoxic medicines focussed on whether they disclose the side effects thereof to their patients and whether they refer their patients to audiologists for screening.

### Participants’ practices with regard to disclosure of ototoxicity

**FIGURE 5:**
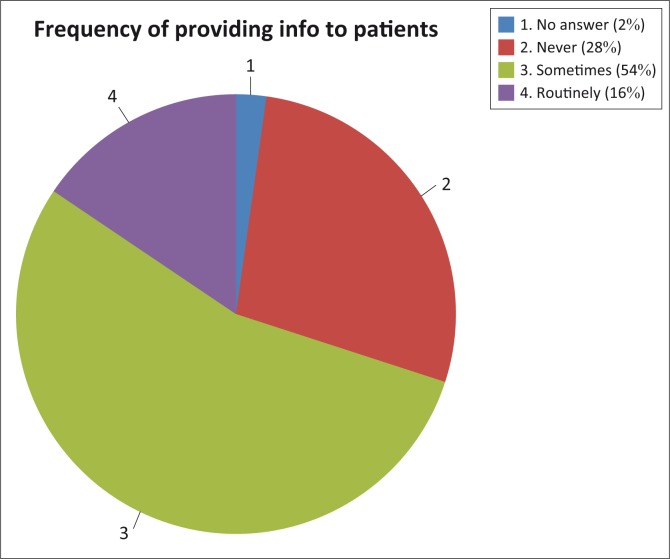
Participants’ practices with regard to disclosure of ototoxicity.

Figure 5 shows the percentage of doctors (*n* = 90) who reportedly have disclosed information on the side effects of the various medicines to their patients. The quantitative results show that only a minority (16%) of the participants routinely disclosed ototoxic risks to their patients, whereas 28% of the participants never disclosed such risks to their patients. The 54% who only ‘sometimes’ disclosed such information did so selectively.

The reasons for non-disclosure of side effects were obtained from open-ended questions that were qualitatively analysed. These results were categorised and are listed in Table 5.

**TABLE 5:** Reasons for non-disclosure and disclosure of side effects of ototoxic medication (qualitative data).

Variable	Items counted	%
**Evidence of disclosure of side effects**		
Doctors show an awareness of side effects	11	12
Doctors tell patients of side effects only in specific con-texts	6	7
Doctors do provide information to patients	3	3
Doctors do tell patients of side effects of only a select group of medicines	22	24
**Evidence of non-disclosure**		
Doctors do not know about side effects	14	15
Doctors do not tell	13	14
Doctors do not consider it a priority to disclose	2	2
Patient are too sick	4	4
Experience time constraints	11	12

Some participants (24%) indicated that they only provide such information for a select group of medications, which could be because they did not know all the medicines that could be ototoxic. This finding was also confirmed by the following statement:

‘Only if patient is on Streptomycin, this other ototoxic drugs are not known by me.’ [P12; Medical officer]

Such results confirm those shown before in Table 5 where there were at least 31% (14% + 17%) of the participants who admitted that they did not have time to discuss otoprotective strategies with their patients. Obtaining informed consent in the proper manner before administering ototoxic medication can be time consuming as it requires engagement with the patient to explain what the treatment will entail, what the risks are and what the alternatives would be (Moodley, 2013c). What makes it even more time consuming is that the doctor has to make sure the client understands his diagnosis and treatment options and then also present the patient with a management plan. The patients must then make an informed choice of whether he or she accepts or declines the doctor’s decision (Moodley, 2013c).

Doctors with a heavy caseload and limited time may find it a challenge to spend so much time on one patient. Audiologists, who are in a supportive role to doctors in the ototoxicity management team, may want to take in upon themselves to disclose the ototoxic risks of certain medicines and obtain informed consent. Considering their patient-centred approach to health care, which is closely related to an EoC perspective, audiologists are likely to regard communication with the patient as an important aspect of health care. They probably have good communication skills because of their professional focus on the management of communication disorders.

Although the majority of the participants (62%) considered otoprotective strategies to be effective, it must be kept in mind that the occurrence of disclosure by doctors may also be context specific. Those doctors working in ICU may have patients who are too sick for discussions about side effects, or they may not be conscious. In the paediatric wards in this particular context, the parents may not be available to discuss the risks.

### Participants’ referral to audiologists

**FIGURE 6:**
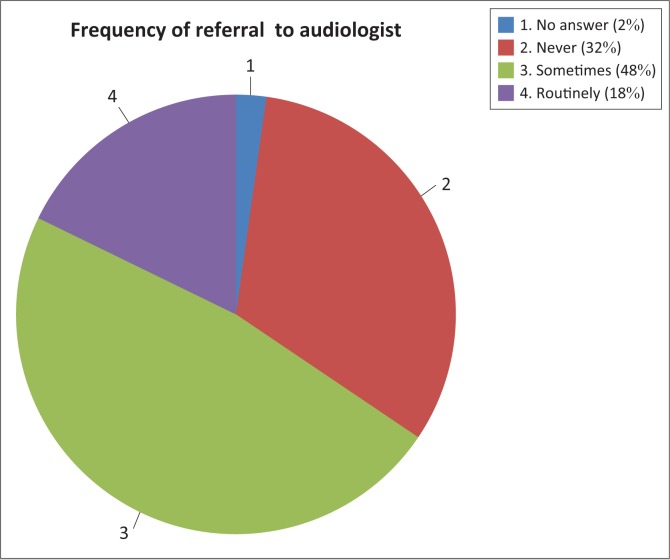
Frequency of referral to audiologists in the management of ototoxicity.

The management of ototoxicity ideally should be based on a team approach, which includes several team members, including an audiologist (Schellack, Wium, Ehlert, Van Aswegen & Gous, 2015). Effective monitoring requires that patients who are on ototoxic medicines be referred to the audiologist for baseline measurements and follow-up monitoring. Figure 6 shows the participants’ reported frequency of referring a patient to the audiology department for screening and monitoring of ototoxicity.

From Figure 6, it can be derived that in this specific hospital it is not common practice to refer patients to an audiologist as only 18% of the participants routinely referred their patients. These findings are consistent with the existing body of knowledge (Wall in Banotai, 2004), which indicates that audiologists are underutilised in hospital settings across the globe. This situation appears to be similar in other contexts in South Africa (De Andrade et al., 2009; Khoza-Shangase, 2013). Examples of some of the reasons provided by the participants for not referring patients are as follows:

‘Due to large amount of patients……. and time constraints.’ [P23; Consultant]

‘There is usually not time to explain’ [P18; Registrar]

The majority of participants reported that they were either too busy or they did not know about the potential ototoxicity of medications and/or the possibility of referral to an audiologist and pharmacist. The lack of referral affects the quality of care and also has serious implications for patients who might develop hearing loss (Professional Board for Speech Language and Hearing Professions, 2015).

### Participants’ practices in obtaining information on ototoxic medicines

In terms of the participants’ knowledge of ototoxicity, the research sought answers as to where the participants obtained their information on medicines (Table 6). The South African Medical Formulator (SAMF), Google and medication insert pamphlets were the information sources most commonly used by all participants across departments or wards. Micromedex (an online compendium of drug information) was least commonly used.

**TABLE 6:** Sources consulted by participants to obtain information on medicines.

	MIMS (%)	Google (%)	Reference books (%)	Academic journals (%)	SAMF (%)	Medication insert pamphlet (%)	Micromedex (%)
Internal Med	8	17	8	6	37	19	6
ICU	12	23	12	12	23	12	8
ENT	12	19	12	15	15	23	4
NICU	17	17	17	8	17	17	8
Paeds	9	24	3	9	42	12	0
Fam Med	9	18	6	8	36	17	6
Full group	10	20	8	9	32	17	5

ENT, ear–nose–throat; Fam Med, family medicine; ICU, intensive care unit; Internal Med, internal medicine; MIMS, Monthly Index of Medical Specialities; NICU, neonatal intensive care unit; Paeds, paediatrics; SAMF, South African Medical Formulator.

**FIGURE 7:**
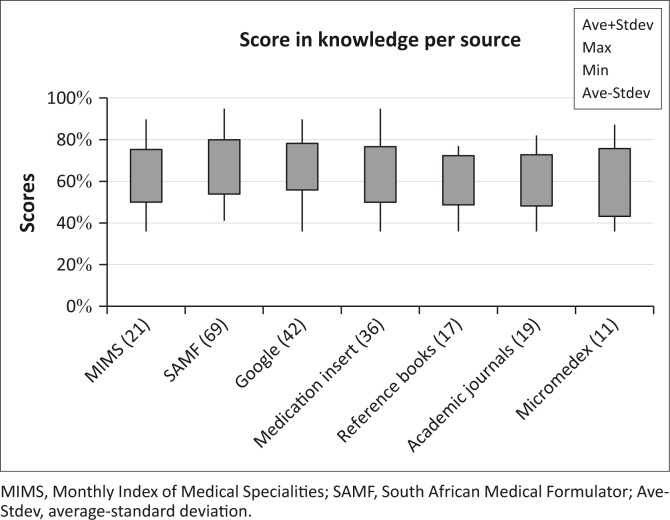
Relationship between scores in knowledge and information sources consulted.

To investigate whether those with higher scores in knowledge consult specific sources of information, a more detailed assessment was carried out to determine the relationship between scores in knowledge and sources consulted (Figure 7). These results show that the average scores of participants were not significantly affected by particular sources of information.

Despite access to such an array of sources, the results indicate that there is room for improvement with regard to their knowledge of ototoxicity. It is possible that because of their heavy workloads, they do not have time to read about ototoxicity and the side effects of certain medication. In the case of serious illness, doctors may not necessarily prioritise the side effects of medicines that are intended to save lives. Should this be the case, it emphasises the need for support for doctors who work with patient groups at risk for ototoxic treatments through CPD activities, which is in line with earlier recommendations by the World Health Organization (1994) to prevent hearing impairment as a result of exposure to ototoxic medication.

### Objective 3: Participants’ opinions on the role of audiologists in the hospital

**FIGURE 8:**
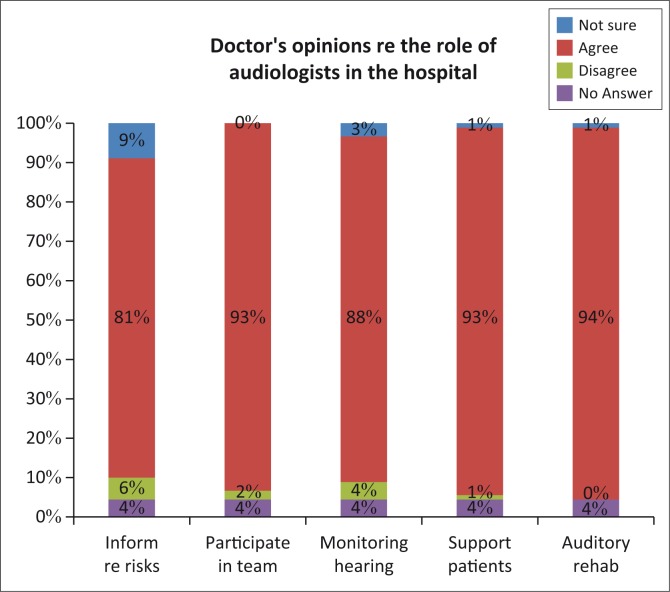
Participants’ perceptions of the role of the audiologist in the hospital.

Because of their inadequate knowledge of ototoxicity, it is possible that the participants were not fully aware of the role of the audiologist. Figure 8 indicates that the participants showed high levels of agreement (81%) towards audiologists having to inform patients of the risks of ototoxic medications. Traditionally, it has been expected of the doctor to disclose the side effects of ototoxic medication prior to starting a patient on such a treatment regimen. Such disclosure is an important element in the process of obtaining informed consent (Moodley, 2013c) and is necessary for ethical practice and effective service delivery. It can be questioned whether audiologists, in a supportive role to the doctor, be the one who can take on the role of disclosing the ototoxicity? Should doctors refer to them timeously before they start treatment, it will be possible to monitor the effect of the medicine on hearing. However, audiologists are mostly consulted only after the patient has been started on the ototoxic medication, which is too late and can be considered a challenge in this context. It may be necessary for audiologists to pertinently promote their role in monitoring ototoxicity so that they get referrals timeously.

The participants strongly agreed (93%) that the audiologist should be part of the ototoxicity management team when patients are prescribed ototoxic medication and that they should monitor hearing ability (88%). Although such findings are in accordance with evidence-based practice, particularly when patients are prescribed platinum chemotherapy agents and aminoglycoside therapy (Banotai, 2004; Professional Board for Speech Language and Hearing Professions, 2015), there are significant differences in the implementation or existence of ototoxicity monitoring protocols across the world (Steffens et al., 2014). In South Africa, Khoza-Shangase (2013) and De Andrade et al. (2009) reported that in none of hospitals included in their studies, were there any audiologists included in the management teams for patients who were on ototoxicity medicines. Careful monitoring will facilitate early identification of ototoxic-induced changes in hearing sensitivity, which will alert doctors and pharmacists to investigate the possibilities for altering the dosage of the prescription, or alternatively changing to a less ototoxic medicine.

The majority of participants (93%) indicated that should patients develop hearing loss, the audiologist should support them. The monitoring by an ototoxicity management team includes audiometry as performed by an audiologist and surveillance of the dose and duration of the treatment by a pharmacist (Schellack et al., 2015; World Health Organization, 1994). Should it be necessary that the current medication be continued, and hearing loss do develop, the patient and/or the family can be counselled and supported (Konrad-Martin et al., 2005). Most of the participants (94%) regarded the audiologist as being responsible for the supply of hearing aids and aural rehabilitation.

Such results show that doctors consider audiologists as important members of the management team. Despite such positive perceptions regarding the audiologists’ role in the hospital, the results also highlight the poor routine referral practices to audiologists, which confirm previous findings by Khoza-Shangase (2013) that reported poor referral to audiologists, and that they were not necessarily known to be part of the team in the treatment of TB. Such poor referral practices could be related to factors such as limited knowledge of ototoxicity and, to a lesser degree, to time restrictions because of high workloads. Should this be the case, then the audiologist has an obligation to actively promote their role in detecting and managing ototoxicity and to consider an increasing supportive role by providing in-service training or CPD activities for doctors in this regard.

### Critical review of the findings

To limit potential bias, the questionnaire was formulated in such a manner as to answer the specific research questions. To increase the face validity of the questionnaire, the questions were presented neatly and in an organised manner. Internal validity was enhanced by including questions that were related to the research objectives and by pilot testing the questionnaire for clarity. Content validity was increased when the questions in the questionnaire were checked by two experts (one audiologist and one pharmacist) in the field of ototoxicity.

A possible threat to the internal validity was the sampling method and the sample size of the study because only doctors who were present at the routine morning meeting on a specific day were included in the research. It is possible that different results would have been obtained if all doctors working in these wards or departments were included. In terms of the threat to external validity, convenience sampling limits the interpretation of results to the specific context and, therefore, the results cannot be generalised to other contexts (Leedy & Ormrod, 2010). Although the analysis of the qualitative data was not confirmed by an independent rater, and thus could have affected quality, this was in part compensated for by triangulating the results. The qualitative data were limited in comparison to the quantitative data and were used to confirm or explain the quantitative results.

Bias was controlled by adhering to ethical guidelines (offering confidentiality in the completion of the questionnaires), and the data collection procedure that allowed the participants to anonymously put their completed questionnaires in a box.

## Conclusion

Patients have a right to make informed decisions about the health care they receive, and doctors have an obligation to inform their patients of adverse effects of the medicines they prescribe. Disclosure of such information is part of ethical practice that has to be integrated in the curricula and professional training of doctors. Failure for participants in this study to disclose such information could be because they were not aware of such risks, and also because South Africa is a multilingual and multicultural context, which has implications on interactions between health professionals and clients, often resulting in ethical dilemmas.

In terms of the main aim of the research, the results showed that doctors in this study need to refresh their knowledge on ototoxicity and could benefit from workshops or CPD activities. With regard to the second objective of this study, it is encouraging to note that the doctors who participated in this study acknowledged these elements of the audiologist’s role, which provides a good foundation for interventions aimed at improving practice with regard to informed decision-making and proper management of ototoxic treatments.

The results from this research emphasised the importance of CPD to make doctors more aware of ethical practices (e.g. such as disclosure of adverse effects of ototoxic medications). The development and presentation of CPD activities that address ethics and ototoxicity should be a collaborative team effort including various disciplines (Banotai, 2004). It is important for doctors to work in a team with other health care professionals (e.g. the audiologists, nurses and pharmacists) to identify early signs of hearing loss and to search for an alternative treatment approach that may cause less harm to hearing.

Whenever there is no other choice than to prescribe ototoxic medication as to save a patient’s life (which often has a high probability of irreversible hearing loss), there are two augmentative ethical perspectives at play. The doctors’ decisions often are based on the principle of causing the least harm and optimum benefit to the patient, which is to save the patient’s life. Audiologists, whose ethical perspective reflects an EoC, strive to be responsive to the patient and the family’s needs and, therefore, play a supportive role. An EoC perspective may require spending more time with the patient and the family and to talk to them about their concerns, which is an expansion of the current roles that were identified by the South African Speech-Language Hearing Association (SASLHA) task team on ototoxicity. Such support entails the provision of information and the development of a jointly agreed upon intervention plan and possibly fitting of hearing aids to address the hearing problem.

The results of this study emphasise the multiple roles required from audiologists in a hospital with regard to the management of ototoxicity. Not only do they have to participate in CPD activities for other health care professionals, but they also should make patients aware of potential risks prior to treatment, monitor the ototoxicity during and after treatment, and support those who do develop hearing loss as a result thereof (Schellack & Naude, 2013). This may be a more extensive role description for audiologists than was originally specified by the Health Professions Council of South Africa (HPCSA). The results of this study emphasise a need for audiologists to expand their roles in the health sector. It is recommended that audiologists in hospital contexts actively advocate their role in the management of ototoxicity to encourage doctors to routinely refer such patients for screening. Future research should investigate how the roles of audiologists in ototoxicity management in South Africa would compare with those internationally.

## Footnotes

1. The terms ‘patient’ and ‘client’ are used interchangeably in this document. The assumption is that patients are those who are sick and admitted to hospital, and clients are not necessarily sick but may suffer from a condition such as hearing or balance problems.

2. Otoprotective strategies: Pharmacokinetics and/or nutrition management to reduce the negative effect of ototoxic medicines on hearing and balance.

## APPENDIX 1

### Ototoxicity: Questionnaire for doctors

**Table:**

articipant’s no:	

#### Section A: Demographic information of doctors

i) Please complete the following:

**Table:**

In which unit/department do you work?	

ii) Please indicate your position in the hospital by marking with an X:

**Table:**

Position	X
Intern	
Medical Officer (MO)	
Registrar	
Consultant	

#### Section B: Ototoxicity – Background/knowledge

1. Please indicate with an X whether the following medication is (a) cochleotoxic, (b) vestibulotoxic, or (c) both (please tick both)?

**Table:**

Medication	(a) Cochleotoxic	(b) Vestibulotoxic
Aspirin		
Ibuprofen		
Indomethacin		
Naproxen (Aleve^®^)		
Diclofenac (e.g. Voltarin^®^, Cataflam^®^ D)		
Mephenamic acid (e.g. Ponstan^®^, Ponac^®^)		
Quinine		
Aminoglycosides		
Gentamycin		
Amikacin		
Tobramycin		
Netilmicin		
Kanamycin		
Streptomycin		
Neomycin		
Macrolides		
Clarithromycin		
Ketolides (Telithromycin)		
Erythromycin (IV)		
Azithromycin		
Glycopeptide		
Vancomycin (IV) (Vancocin CP^®^)		
Peptide (Capreomycin)		
Antineoplastic agents:		
Platinum compounds (e.g. Cisplatin, Carboplatin)		
Iron-Chelating (e.g. Deferoxamine)		
Vinca Alkaloids (e.g. Vincristine, Vinblastine, Vinorel-bine)		
Nitrogen Mustard analogues		
Cyclophosphamide, Chlorambucil, Melphalan, Ifos-famide		
Loop Diuretics (e.g. Furosemide: Lasix^®^, Puresis^®^, Beurises^®^, Uretic^®^)		

2. Are there any other possible side effects, other than cochleotoxicity and vestibulotoxicity, with the use of ototoxic medication? If yes, please state these possible side effects.
  ___________________________________________________________________________________________________
  ___________________________________________________________________________________________________
  ___________________________________________________________________________________________________
  ___________________________________________________________________________________________________

**Table:**

**3. Which otoprotective strategies can you recommend when a patient is on any ototoxic medication?**

**Table:**

**4. What would you consider as risk factors for the predisposi-tion of patients to ototoxicity?Please mark all relevant options with an X:**	**Mark with X**
4.1. Very young age	
4.2. Very old age	
4.3. Infection	
4.4. Hydration status	
5. Dose, duration and mode of administration	
6. Renal insufficiency or insult	
7. Hepatic failure	
8. Metastasis	

5. Please indicate by marking with an X which of the following conditions do you consider to be at greater risk of **loop diuretic-**induced ototoxicity? (E.g. furosemide (Lasix), torasemide, bumetanide).

**Table:**

Condition	Mark with X
Renal impairment	
Seizures	
Premature infants	
Concomitant use of aminoglycoside antibiotics	
Peptic ulcer disease	
Cardiac failure	

#### Section C: Current practices of doctors

6. Do you consult any of the following sources of information to determine if medication is cochleotoxic or vestibulotoxic before prescribing it? Please mark all relevant options with an X.

**Table:**

Reference	Mark with X
MIMS (Monthly Index of Medical Specialities)	
SAMF (South African Medicines Formulary)	
Google	
Medication insert pamphlet	
Reference books	
Academic Journals	
Micromedex^®^ (online resource)	

7. How often do you **refer patients** who are on ototoxic medication to an audiologist for a hearing evaluation? Please mark with an X.

**Table:**

Never	Sometimes	Routinely
		

8. How often do you **provide patients with information** re the potential side effects of ototoxic medicine before prescribing such medications?

**Table:**

Never	Sometimes	Routinely
		

9. Please explain your answer to Question no 8?
  ___________________________________________________________________________________________________
  ___________________________________________________________________________________________________
  ___________________________________________________________________________________________________
10. To what extent do you agree with the following statements concerning otoprotective strategies? Please indicate with an X.

**Table:**

	Disagree	Agree	Not sure
10.1 I do not consider them to be effective			
10.2 I do not have time to include them in the regi-men			
10.3 It is a waste of time			
10.4 I consider them to be effective			
10.5 Otoprotective strategies should be adhered to whenever possible			
10.6 I would like to know more about ototoxicity and otopro-tective strategies			

#### Section D: Role of the audiologist in the hospital

11. Considering the role of the audiologist in the hospital, what is your opinion in terms of the following statements? Please mark with an X.

**Table:**

The audiologist should:	Disagree	Agree	Not sure
11.1 Inform patients (and /or guardians and significant others) with regard to the risks of ototoxic medication			
11.2 Be part of the multi-disciplinary team in the treatment of patients			
11.3 Be responsible for monitoring hearing during treatment with ototoxic drugs			
Support the patient who develops hearing loss as a result of ototoxicity			
Provide the patient with hearing aids and rehabilita-tion			

#### Section E: Information dissemination

12. What type of information with regard to *ototoxic medication* do you think should be provided to patients in a hospital?
13. In your opinion, what *other information* should be included in an information pamphlet to be given to patients who are placed on ototoxic medication? Please provide your suggestions in bullet style.
  ___________________________________________________________________________________________________
  ___________________________________________________________________________________________________
  ___________________________________________________________________________________________________

Thank you for your time!

## References

[CIT0001] Banotai A (2004). Ototoxicity hearing loss and pharmacology. Advance Healthcare Network for Speech and Hearing, 14(2), 10.

[CIT0002] Beauchamp T.L., & Childress J.F (2013). Principles of biomedical ethics (7th edn.). New York: Oxford University Press.

[CIT0003] Cheng A.G., Huth M.E., & Ricci A.J (2011). Mechanisms of Aminoglycoside ototoxicity and target of hair cell protection. International Journal of Otolaryngology, 1(19), 1–19. 10.1155/2011/937861 PMC3202092 22121370

[CIT0004] Creswell J.W (2008). Mixed methods research. Paper presented at the Faculty of Education centenary celebratory lecture, Pretoria.

[CIT0005] De Andrade V., Hajat F., & Khoza-Shangase K (2009). Perceptions of oncologists at two state hospitals in Gauteng regarding the ototoxic effects of cancer chemotherapy: A pilot study. African Journal of Pharmacy and Pharmacology, 3(6), 307–318.

[CIT0006] Dobie R.A., Black F.O., Pezsnecker S.C., & Stallings V.L (2006). Hearing loss in patients with vestibulotoxic reactions to gentamicin therapy. Archives of Otolaryngology – Head & Neck Surgery, 132(3), 253–257. http://dx.doi.org/10.1001/archotol.132.3.253 16549744 10.1001/archotol.132.3.253

[CIT0007] Emanuel E.J., Grady C., Crouch R.A., Lie R.K., Miller F.G., & Wendler D. (Eds.) (2008). The Oxford textbook of clinical research ethics. New York: Oxford University Press.

[CIT0008] Engler D., Schellack N., & Naude A (2013). Use of amikacin in neonates and related ototoxicity. Professional Nursing Today, 17(1), 24–27.

[CIT0009] Gerber B (2013). Identity and discourse: A critical philosophical investigation of the influence of the intellectual self-image of the medical profession on communicatively effective care to patients. D. Phil dissertation, Stellenbosch: University of Stellenbosch.

[CIT0010] Gerteis M., Edgman-Levitan S., Daley J., & Delbanco T (1993). Through the patient’s eyes: understanding and promoting patient-centered care. San Francisco, CA: Jossey-Bass.

[CIT0011] Gilligan C (1982). In a different voice: Psychological theory and women’s development. Cambridge, MA: Harvard University Press.

[CIT0012] Hall J.W (2000). Handbook of otoacoustic emissions. Stamford, CT: Thomson Learning.

[CIT0013] Health Professions Council of South Africa (2005). Exit level outcomes. Pretoria: HPCSA.

[CIT0014] Hellberg V., Wallin I., Eriksson S., Hernlund E., .Jerremalm E., Berndtsson, et al (2009). Cisplatin and oxaliplatin toxicity: Importance of cochlear kinetics as a determinant for ototoxicity. Journal of the National Cancer Institute, 101(1), 37–47. http://dx.doi.org/10.1093/jnci/djn418 19116379 10.1093/jnci/djn418 PMC2639295

[CIT0015] Katz J., Medwetsky L., Burkard R., & Hood L (2009). Handbook of clinical audiology (6th edn.). New York: Lippincott Williams & Williams.

[CIT0016] Khoza-Shangase K (2013). Ototoxicity in tuberculosis treatment in South Africa: Exploring the current status. African Journal of Pharmacy and Pharmacology, 7(30), 2140–2145. http://dx.doi.org/10.5897/AJPP12.722

[CIT0017] Kirk L.M (2007). Professionalism in medicine: Definitions and considerations for teaching. Baylor University Medical Center Proceedings, 20(1), 13–16. 17256035 10.1080/08998280.2007.11928225 PMC1769526

[CIT0018] Konrad-Martin D., Gordon J.S., Reavis K.M., Wilmington D.J., Helt W.J., & Fausti S.A (2005). Audiological monitoring of patients receiving ototoxic drugs. ASHA Division 6: Perspectives on Hearing and Hearing Disorders: Research and Diagnosis, 9(1), 17–22. http://dx.doi.org/10.1044/hhd9.1.17

[CIT0019] Leedy P.D., & Ormrod J.E (2010). Practical research: Planning and design (9th edn.). Boston, MA: Pearson.

[CIT0020] Moodley K (2013a). A place for ethics, law and human rights in health care. In Moodley K. (Ed.), Medical ethics, law and human rights (3rd edn., pp. 3–5). Pretoria: Van Schaik.

[CIT0021] Moodley K (2013b). Professionalism. In Moodley K. (Ed.), Medical ethics, law and human rights: A South African perspective (3rd edn, pp. 143–155). Pretoria: Van Schaik.

[CIT0022] Moodley K (2013c). Respect for patient autonomy. In Moodley K. (Ed.), Medical ethics, law and human rights - A South African perspective (3rd edn, pp. 41–55). Pretoria: Van Schaik.

[CIT0023] Naude A.M., & Bornman J (2014). A systematic review of ethics knowledge in audiology (1980–2010). American Journal of Audiology, 23, 151–157. http://dx.doi.org/10.1044/2014_AJA-13-0057 24695796 10.1044/2014_AJA-13-0057

[CIT0024] Paul R., & Norbury C (2012). Language disorders from infancy through adolescence: Listening, speaking, reading, writing and communicating. New York: Elsevier Health Sciences.

[CIT0025] Professional Board for Speech Language and Hearing Professions (2015). Guidelines for audiological management of patients on treatment that includes ototoxic medications. In HPCSA (Ed.) Pretoria: HPCSA.

[CIT0026] Roland P.S., & Rutka J.A (2004). Ototoxicity. London: B.C. Decker.

[CIT0027] Schellack N., & Naude A.M (2013). An overview of pharmacotherapy-induced ototoxicity. South African Family Practice, 55(4), 357–365. http://dx.doi.org/10.1080/20786204.2013.10874377

[CIT0028] Schellack N., Wium A.M., Ehlert K., Van Aswegen Y., & Gous A (2015). Establishing a pharmacotherapy induced ototoxicity programme within a service-learning approach. South African Journal of Communication Disorders, 62(1), E1–E8. http://dx.doi.org/16.4102/sajcd.V62i!110 10.4102/sajcd.v62i1.96 PMC5843252 26304216

[CIT0029] Selimoglu E (2013). Aminoglycoside-induced ototoxicity. Current Pharmaceutical Design, 13(1), 119–126. http://dx.doi.org/10.2174/138161207779313731 10.2174/138161207779313731 17266591

[CIT0030] South African Government Gazette (2005). Children’s amendment act. Pretoria: Juta and Company.

[CIT0031] Stach B.A (2010). Clinical audiology: An introduction (2nd edn.). New York: Delmar, Cengage Learning.

[CIT0032] Steffens L., Venter K., O’Beirne G.A., Kelly-Campbell R., Gibbs D., & Bird P (2014). The current state of ototoxicity monitoring in New Zealand. The New Zealand Medical Journal, 127(1398), 85–97. 25146864

[CIT0033] Van Niekerk A.A (2013). Ethics theories and the principlist approach in bioethics. In Moodley K. (Ed.), Medical ethics, law and human rights: A South African perspective (3rd edn.). Pretoria: Van Schaik.

[CIT0034] World Health Organization (1994). Report of an informal consultation on strategies for prevention of hearing impairment from ototoxic drugs. Geneva: World Health Organization.

[CIT0035] Yansey A., Harris M.S., Egbelakin A., Gilbert J., Pisone D.B., & Renbarger J (2012). Risk factors for cisplatin associated ototoxicity in paediatric oncology patients. Pediatric Blood Cancer, 59(1), 144–148. http://dx.doi.org/10.1002/pbc.24138 22431292 10.1002/pbc.24138 PMC3767972

